# Functional outcomes with esketamine in treatment-resistant depression: A 6-month multicenter real-world study

**DOI:** 10.1192/j.eurpsy.2026.12233

**Published:** 2026-06-10

**Authors:** Riccardo Guglielmo, Miriam Olivola, Alberto Inuggi, Elisa Cavanna, Elisa Briasco, Beatriz Pereira Da Silva, Andrea Escelsior, Gabriele Giacomini, Giovanni Martinotti, Bernardo Maria Dell’Osso, Mario Amore, Gianluca Serafini

**Affiliations:** 1 University of Genoa: Università degli Studi di Genova, Italy; 2 IRCCS Ospedale Policlinico San Martino, Italy; 3 University of Pavia: Università degli Studi di Pavia, Italy; 4 Università degli Studi Gabriele d’Annunzio Chieti Pescara, Italy; 5 Università degli Studi di Milano, Italy

**Keywords:** esketamine, functional remission, real-world study, treatment-resistant depression, long-term outcomes

## Abstract

**Background:**

Esketamine has demonstrated efficacy in treatment-resistant depression (TRD), but medium-term real-world functional outcomes remain understudied. This study described 6-month depressive symptoms and functional trajectories during routine esketamine treatment, focusing on functional outcomes and remission correlates.

**Methods:**

In this prospective multicenter study, 60 patients with TRD initiating intranasal esketamine augmentation were assessed at baseline, Month 1, Month 3, and Month 6 using the Montgomery-Åsberg Depression Rating Scale (MADRS) and Sheehan Disability Scale (SDS). Mixed-effects models, Turnbull interval-censored time-to-remission analysis, logistic regression, and ROC analyses were performed.

**Results:**

MADRS and SDS improved significantly at all follow-up time points (*p* < 0.001). At Month 6, symptomatic response and remission rates were 78.3 and 46.7%, while functional response and remission rates were 78.3 and 33.3%, respectively. Turnbull estimates showed cumulative functional remission rates of 5.0, 15.0, and 33.3% at Months 1, 3, and 6, respectively. MADRS-SDS correlations decreased over time, supporting partial symptomatic-functional dissociation. Higher baseline SDS (OR = 0.73, 95% CI: 0.59–0.89) and more previous antidepressant trials (ADTs; OR = 0.53, 95% CI: 0.35–0.82) were associated with lower odds of Month 6 functional remission; bootstrap internal validation supported coefficient stability. ROC-derived thresholds were considered sample-dependent and not clinically validated. Cumulative esketamine dosage was associated with functional response but not remission.

**Conclusions:**

Depressive symptoms and functioning improved progressively over 6 months during esketamine treatment in routine clinical care. Functional remission followed a slower trajectory than symptomatic remission and had partly distinct correlates, supporting functional monitoring as a distinct TRD outcome.

## Introduction

Treatment-resistant depression (TRD) is commonly operationalized as an insufficient response to at least two antidepressant trials (ADTs) of adequate dose and duration within the same major depressive episode (MDD), although no universally accepted definition exists [1]. TRD is associated with persistent symptom severity, poorer prognosis, increased suicidality, and marked psychosocial impairment. Yet symptomatic improvement alone often misses meaningful recovery, as patients continue to experience substantial disability despite symptom reduction.

Esketamine has emerged as an important treatment option for adults with TRD, with randomized controlled trials and maintenance studies supporting its efficacy in reducing depressive symptoms and preventing relapse [2, 3]. However, the evidence base has been predominantly symptom-centered, whereas recovery also requires restoration of everyday functioning and reengagement in social and occupational roles. This distinction is clinically relevant because symptomatic response and functional recovery are only partially overlapping, and the latter may follow a different, and often slower, trajectory than symptom change alone.

Within the available esketamine literature, functioning has generally been assessed as a secondary, exploratory, or post hoc outcome rather than as a central focus of investigation. Early randomized evidence suggested potential improvement in functional burden, although between-group differences were not always statistically robust [4]. Subsequent phase III and post hoc analyses further supported beneficial effects on functioning during the acute phase, particularly within the first weeks of treatment [5, 6]. More recently, in ESCAPE-TRD, esketamine was associated with greater functional improvement and the likelihood of functional remission compared with active control over 32 weeks [7]. A recent review similarly concluded that evidence for beneficial effects on functional impairment is growing, but remains considerably less developed than the literature focused on symptom outcomes [8].

At the same time, important gaps remain. Much of the currently available evidence derives from acute-phase trials, secondary analyses, or controlled study settings, whereas multicenter real-world data specifically characterizing medium-term functional trajectories remain limited. Naturalistic cohorts better reflect the heterogeneity and comorbidity burden of patients seen in routine care, and relatively little is known about which baseline characteristics distinguish patients who achieve remission, particularly functional remission, from those who do not [9].

In this context, the present multicenter real-world study aimed to describe the 6-month clinical and functional course among patients with TRD who initiated esketamine treatment in routine care. We also examined the relationship between symptomatic and functional outcomes over time and explored whether baseline sociodemographic and clinical characteristics could help distinguish patients who achieved remission at 6 months from those who did not. By combining a multicenter naturalistic design, medium-term follow-up, and an explicit focus on remission and baseline predictors, this study seeks to extend current evidence beyond acute-phase efficacy and to provide a more clinically relevant characterization of recovery during esketamine treatment.

## Methods

### Setting and design

This was a multicenter, longitudinal, real-world observational study conducted at outpatient psychiatric centers at the University General Hospital “San Martino” in Genoa and at ASST Pavia. Patients were enrolled between 2023 and 2025. Esketamine treatment was prescribed and administered in routine clinical practice, and no experimental allocation of treatment was performed.

### Sample

Patients aged 18 years and older were included if they met DSM-5 criteria for MDD or bipolar disorder (BD) in a current depressive episode [10], fulfilled operational criteria for TRD [1, 11], and were initiating intranasal esketamine augmentation in routine clinical care.

Esketamine was administered according to approved prescribing information and local clinical practice. The standard schedule included an induction phase with two administrations per week for 4 weeks, followed by a maintenance phase in which treatment frequency was individualized. During maintenance, administrations could be scheduled twice weekly, once weekly, or every 2 weeks according to clinical response, tolerability, clinician judgment, and patient preference. Esketamine dose was flexibly adjusted within the approved dosing range, mainly on the basis of efficacy and tolerability. Patients received concomitant standard clinical care, including pharmacotherapy and, when applicable, psychotherapy. Concomitant psychotropic medications were prescribed and adjusted by treating clinicians according to individual clinical need and were not experimentally controlled. Baseline use of major concomitant medication classes, including antipsychotics, mood stabilizers, and benzodiazepines, as well as ongoing psychotherapy, was recorded.

### Variables and measures

Clinical and functional assessments were collected at baseline (M0), Month 1 (M1), Month 3 (M3), and Month 6 (M6). Depressive symptom severity was assessed using the Montgomery-Åsberg Depression Rating Scale (MADRS) [12], whereas functional impairment was assessed using the Sheehan Disability Scale (SDS) total score [13]. Personality disorder (PD) comorbidity was clinically assessed at baseline by the treating psychiatrists at the participating tertiary centers, according to DSM-5 clinical criteria. No structured PD interview or specific diagnostic instrument for PD was systematically administered as part of the study protocol. For descriptive purposes, PD diagnoses were grouped according to DSM-5 cluster classification when a specific cluster diagnosis was available. Patients without a specified cluster diagnosis were classified as having other specified/unspecified PD. The primary outcome was longitudinal change in functional impairment over 6 months. Secondary outcomes included longitudinal change in depressive symptom severity, symptomatic and functional response and remission, time to first functional remission, baseline and treatment-related correlates of Month 6 outcomes, and predictive performance of baseline clinical variables.

Symptomatic response was defined as a reduction of at least 50% from baseline in MADRS total score, and symptomatic remission as MADRS ≤10 [14]. Functional response was defined as a reduction of at least four points from baseline in SDS total score (sdsresp), while a reduction of at least eight points was additionally examined as a more stringent functional response threshold (sdsresp2) [15]. Functional remission was defined as SDS ≤ 6 [16]. However, given that this cutoff was originally validated in MDD and generalized anxiety disorders clinical trial populations, descriptive sensitivity analyses were performed at M6 using a stricter threshold (SDS ≤ 5) and a more permissive low-disability threshold (SDS ≤ 8). Time to first functional remission was defined as the interval between treatment initiation and the first follow-up visit at which SDS remission criteria were met. Because assessments were performed at discrete time points, event time corresponded to the first observed visit at which remission criteria were met.

### Statistical analysis

Continuous variables were summarized as mean and standard deviation (SD) or median and interquartile range (IQR), as appropriate, whereas categorical variables were described as counts and percentages.

Longitudinal changes in MADRS and SDS were examined using mixed-effects models with time as a categorical fixed effect and participant as a random intercept. Models were fitted in the analytic cohort of patients who initiated esketamine treatment and had complete MADRS and SDS assessments from baseline to Month 6; no outcome imputation was performed. Post hoc power analyses were conducted for longitudinal MADRS and SDS changes using the achieved sample size and four repeated assessments. Dosage was examined as a time-varying covariate, including a time × dosage interaction. Cumulative dosage was calculated as the sum of the actual esketamine doses administered during each follow-up interval: baseline to M1, M1 to M3, and M3 to M6. Pairwise comparisons versus baseline were FDR (False Discovery Rate)-corrected.

Associations between MADRS and SDS change scores were examined using Spearman correlations. Because functional remission was assessed only at scheduled visits, time to first functional remission was treated as interval-censored, and cumulative remission probabilities were estimated using the Turnbull estimator. Baseline correlates of Month 6 outcomes were explored using Mann–Whitney *U* tests and chi-square or Fisher’s exact tests, with FDR correction. Treatment initiators and non-initiators were compared descriptively using Mann–Whitney *U* and Fisher’s exact tests. PD was analyzed as a binary clinical comorbidity variable because cluster-level sample sizes were small.

Variables showing nominal evidence of association were entered into logistic regression models. Secondary functional response models included the baseline variable retained from univariate screening. For Month 6 functional remission, the final prognostic model was restricted to baseline SDS and ADTs to preserve parsimony, given the limited number of remission events; bootstrap internal validation with 1,000 resamples assessed coefficient stability. ROC analyses were performed for functional remission, and ROC-derived thresholds from univariate ROC analyses of baseline SDS and ADTs were evaluated using leave-one-out cross-validation and interpreted as sample-dependent estimates.

### Ethics approval and trial registration

The study was conducted in accordance with the Declaration of Helsinki and applicable regulatory requirements. All patients provided written informed consent. Study protocols and amendments were approved by the relevant local Ethics Committees at each participating site: Genoa (Opinion # 2025/12) and Pavia (Opinion # 84157/21; amendment # 0102231/21). The study was registered on ClinicalTrials.gov (Identifier: NCT07146503).

## Results

### Sample characteristics

**Figure 1.**
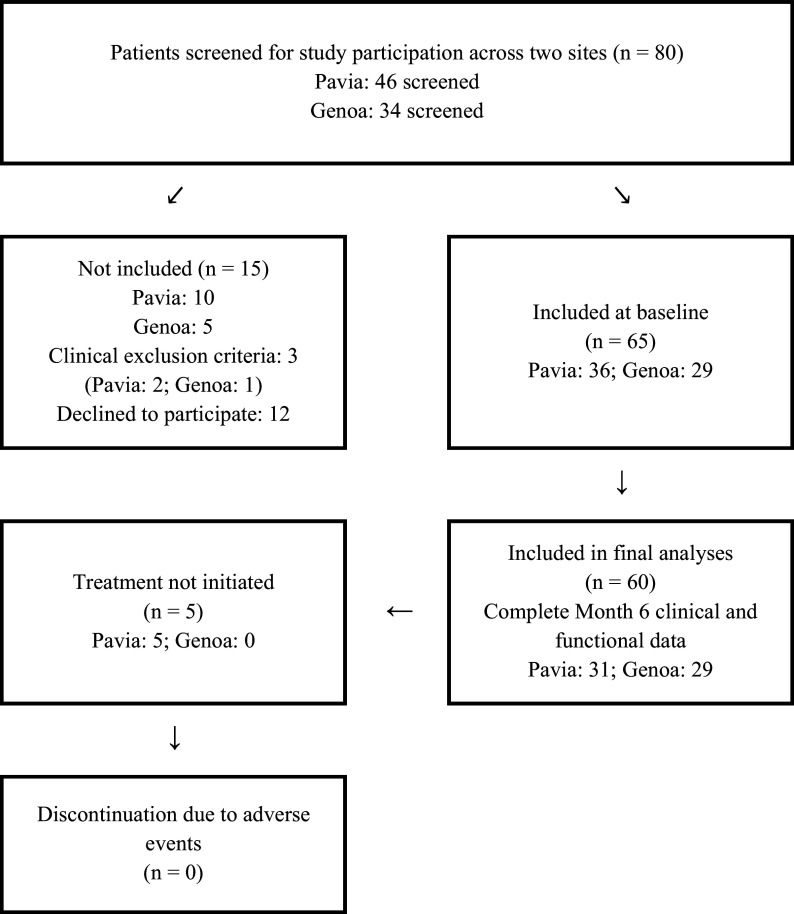
Study flow diagram. Flow diagram of patient screening, baseline assessment, treatment initiation, and final analytic sample across the two participating sites. A total of 80 patients were screened (Pavia, *n* = 46; Genoa, *n* = 34), 65 met eligibility criteria and had baseline data recorded, and 60 initiated esketamine treatment and had complete Month 6 clinical and functional assessments. Five patients had baseline data recorded but did not initiate treatment and were therefore excluded from longitudinal treatment outcome analyses. No discontinuations due to adverse events were recorded. Figure 1. long description.

Of 80 screened patients, 65 met eligibility criteria and had baseline data recorded. Five patients did not initiate treatment and were excluded from longitudinal analyses. The final analytic sample included 60 treatment initiators with complete Month 6 clinical and functional assessments (Figure 1). Baseline characteristics are reported in Table 1, and the comparison between treatment initiators and non-initiators is reported in Supplementary Table S1. The sample had a mean age of 49.8 years, 63.3% were female, 75.0% had MDD, and 25.0% had BD in a current depressive episode. PD comorbidity was present in 32 patients (53.3%): 3 Cluster A, 9 Cluster B, 8 Cluster C, and 12 other specified/unspecified PD.

**Table 1.** Sociodemographic and clinical characteristics of the study sample at baseline Table 1. long description.

Variable	Value
Participants, *n*	60
Age, years, mean (SD)	49.8 (15.9)
Age range, years	19–80
Female sex, *n* (%)	38 (63.3)
University degree, *n* (%)	16 (26.7)
Married, *n* (%)	33 (55.0)
Employment status, *n* (%)	
Employed	33 (55.0)
Unemployed	12 (20.0)
Retired	15 (25.0)
Diagnosis, *n* (%)	
Major depressive disorder	45 (75.0)
Bipolar disorder, current depressive episode	15 (25.0)
Functional impairment at baseline, mean (SD)	21.4 (4.9)
Depressive symptom severity at baseline, mean (SD)	34.2 (7.1)
Duration of current depressive episode, months, median [IQR]	12 [6–18]
Duration of mood disorder, years, median [IQR]	20 [9–28]
Previous pharmacological trials, median [IQR]	5 [3–6]
Baseline psychiatric medications, *n* (%)	
Antipsychotics	29 (48.3)
Mood stabilizers	22 (36.7)
Benzodiazepines	27 (45)
Median no. psychopharmacological agents, median [IQR]	3 [2–4]
Ongoing psychotherapy, *n* (%)	19 (31.7)
Psychiatric comorbidity, *n* (%)	
Personality disorder	32 (53.3)
Obsessive-compulsive disorder	1 (1.6)
Eating disorder	1 (1.6)
Anxiety disorder	1 (1.6)

*Note:* Data are presented as mean (SD), median [interquartile range, IQR], or *n* (%), as appropriate. Baseline refers to the assessment performed prior to esketamine treatment initiation. IQR, interquartile range; SD, standard deviation. Among the 32 patients with personality disorder comorbidity, 3 were classified as Cluster A, 9 as Cluster B, 8 as Cluster C, and 12 as having other specified/unspecified personality disorder.

### Longitudinal clinical and functional outcomes

**Figure 2.**
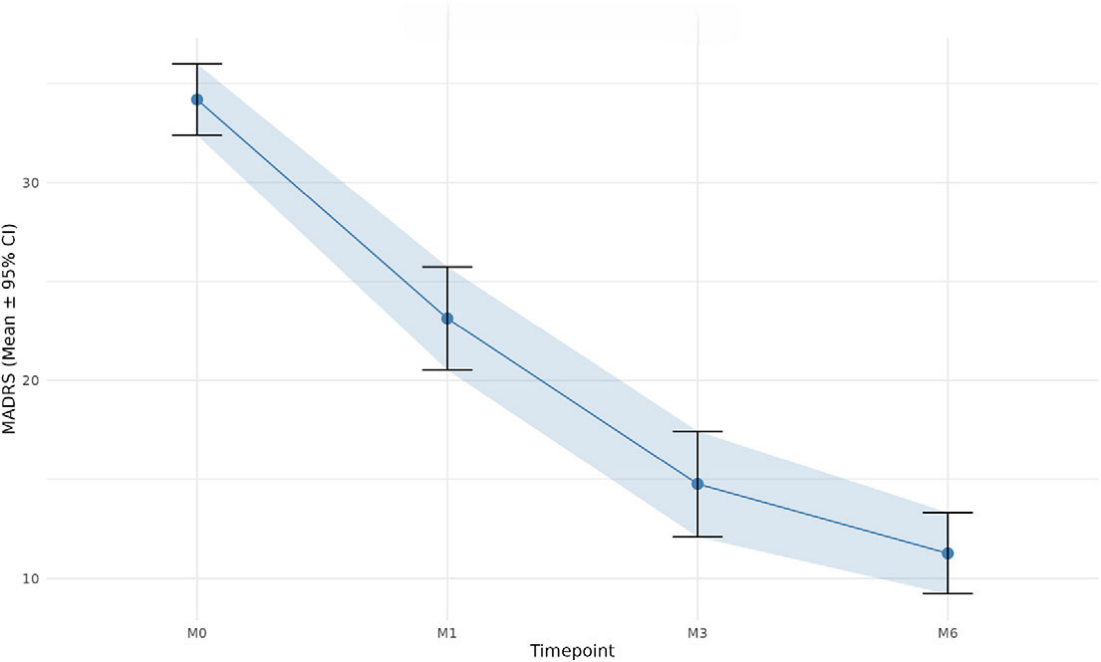
Longitudinal change in depressive symptom severity over follow-up. Mean MADRS total scores at baseline (M0), Month 1 (M1), Month 3 (M3), and Month 6 (M6) in the analytic sample. Points represent observed means, and error bars indicate 95% confidence intervals. MADRS scores decreased progressively over follow-up, consistent with the significant time effect observed in mixed-effects models.

**Figure 3.**
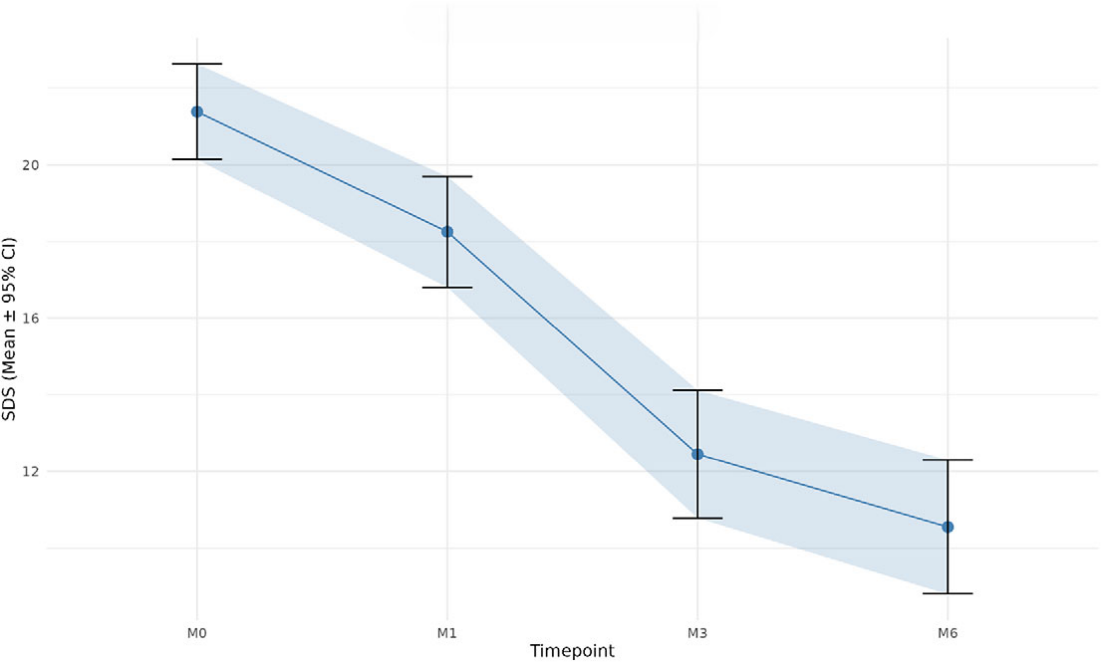
Longitudinal change in functional impairment over follow-up. Mean SDS total scores at baseline (M0), Month 1 (M1), Month 3 (M3), and Month 6 (M6) in the analytic sample. Points represent observed means, and error bars indicate 95% confidence intervals. SDS scores decreased progressively over follow-up, consistent with the significant time effect observed in mixed-effects models. Figure 3. long description.

Both depressive symptoms and functional impairment improved significantly over time (Tables 2 and 3). Mean MADRS decreased from 34.2 ± 7.1 at baseline to 23.1 ± 10.3 at Month 1, 14.8 ± 10.5 at Month 3, and 11.3 ± 8.1 at Month 6; symptomatic response rates were 30.0% at Month 1, 68.3% at Month 3, and 78.3% at Month 6; and symptomatic remission rates were 10.0, 30.0, and 46.7%, respectively. Mean SDS decreased from 21.4 ± 4.9 at baseline to 18.3 ± 5.7 at Month 1, 12.5 ± 6.6 at Month 3, and 10.6 ± 6.9 at Month 6; sdsresp rates were 48.3, 78.3, and 78.3%, respectively; sdsresp2 rates were 3.3% at Month 1, 70.0% at Month 3, and 66.7% at Month 6; functional remission rates were 5.0, 15.0, and 33.3%, respectively. At Month 6, sensitivity analyses using alternative SDS thresholds showed rates of 31.7% for SDS ≤ 5, 33.3% for SDS ≤ 6, and 45.0% for SDS ≤ 8 (Supplementary Table S2). Mixed-effects models confirmed a highly significant effect of time for both MADRS and SDS, with all post hoc comparisons versus baseline remaining significant after FDR correction (Figures 2 and 3). Post hoc power analyses indicated observed standardized effect sizes of 0.452 for SDS and 0.426 for MADRS. The estimated power was 86.9% for SDS and 82.7% for MADRS, with minimum detectable effect sizes of 0.485 and 0.469, respectively, under the assumed repeated-measures correlation structure (Supplementary Table S3). When dosage was added as a time-varying covariate, higher dosage was significantly associated with lower MADRS and lower SDS, whereas the time × dosage interaction was not significant for either outcome, indicating a stable association between treatment exposure and improvement across follow-up. Changes in MADRS and SDS were moderately correlated over time, with rho = 0.653 at M1, rho = 0.635 at M3, and rho = 0.571 at M6 (all *p* < 0.001), supporting partial but incomplete overlap between symptomatic and functional improvement.

**Table 2.** Change in MADRS total score over time Table 2. long description.

Time	Mean	SE	95% CI	*p*-value versus M0
M0	34.200	0.917	32.40–36.00	–
M1	23.100	1.330	20.49–25.71	<0.001
M3	14.800	1.356	12.14–17.46	<0.001
M6	11.300	1.046	9.25–13.35	<0.001

*Note:* Data are presented as observed mean, standard error (SE), and 95% confidence interval (CI). Pairwise *p*-values refer to post hoc comparisons versus baseline (M0) from mixed-effects models and were FDR-corrected. MADRS, Montgomery–Åsberg Depression Rating Scale.

**Table 3.** Change in SDS total score over time Table 3. long description.

Time	Mean	SE	95% CI	*p*-value versus M0
M0	21.400	0.633	20.16–22.64	–
M1	18.300	0.736	16.86–19.74	<0.001
M3	12.500	0.852	10.83–14.17	<0.001
M6	10.600	0.891	8.85–12.35	<0.001

*Note:* Data are presented as observed mean, standard error (SE), and 95% confidence interval (CI). Pairwise *p*-values refer to post hoc comparisons versus baseline (M0) from mixed-effects models and were FDR-corrected. SDS, Sheehan Disability Scale.

### Exploratory analyses by diagnostic polarity

Exploratory analyses by diagnostic polarity compared patients with MDD (*n* = 45) and BD in a current depressive episode (*n* = 15). MADRS improved in both groups, with no significant diagnosis × time interaction (MDD: −3.61; BD: −3.13 points per time point; *p* = 0.37). SDS also improved in both groups, but the diagnosis × time interaction was significant (*p* = 0.0014; likelihood-ratio test *p* = 0.0013), indicating slower functional improvement in BD (MDD: −2.00; BD: −1.18 points per time point). These findings were interpreted cautiously, given the small BD subgroup.

### Functional remission over time

**Figure 4.**
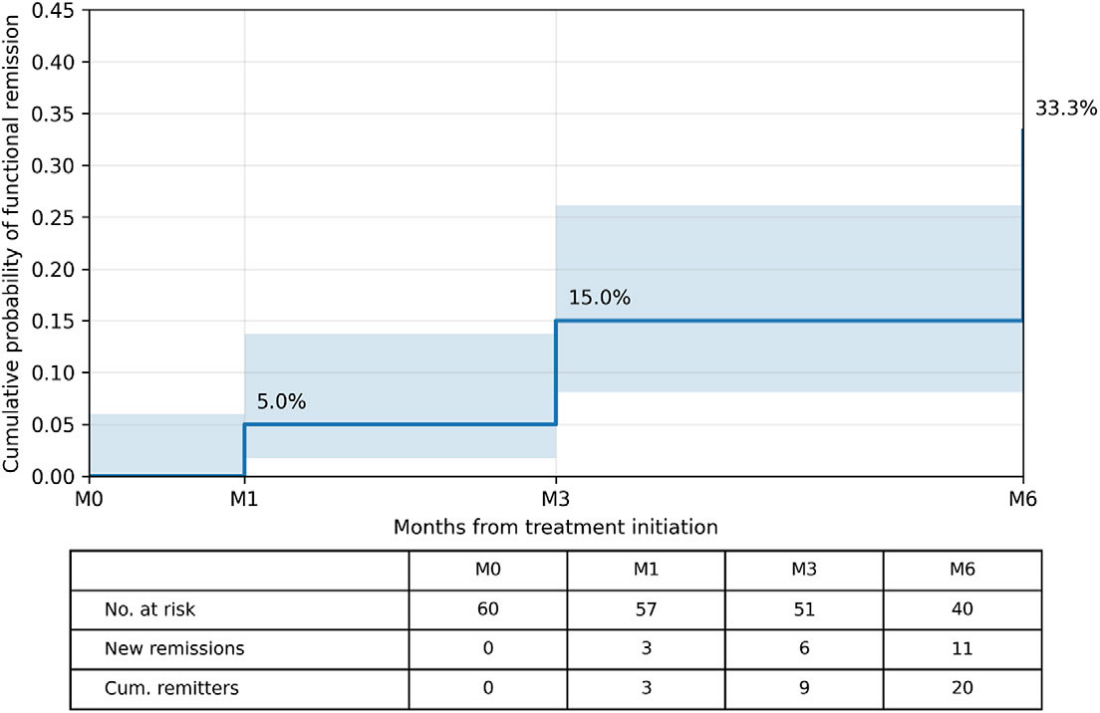
Turnbull interval-censored estimate of time to first functional remission. Cumulative probability of first observed functional remission over 6 months, estimated using the Turnbull method for interval-censored data. Functional remission was defined as an SDS total score ≤ 6. Cumulative remission rates were 5.0% at Month 1, 15.0% at Month 3, and 33.3% at Month 6. Shaded bands represent 95% confidence intervals. The table reports numbers at risk, newly observed first remissions, and cumulative remitters. The median time to first functional remission was not reached. Figure 4. long description.

Using the Turnbull estimator for interval-censored data, cumulative functional remission rates were 5.0% at Month 1, 15.0% at Month 3, and 33.3% at Month 6. Newly observed first remissions were 3 between baseline and Month 1, 6 between Month 1 and Month 3, and 11 between Month 3 and Month 6. Because fewer than half of patients achieved functional remission within the 6-month observation period, the median time to first functional remission could not be estimated (Figure 4).

### Baseline correlates of Month 6 outcomes

In univariate comparisons, no baseline variable significantly distinguished MADRS responders from nonresponders, although ADTs showed a trend-level association (*p* = 0.085). Likewise, no robust baseline discriminator emerged for MADRS remission, although duration of the current depressive episode showed a marginal association (*p* = 0.056). For sdsresp, baseline symptom and disability severity did not differ between responders and nonresponders, whereas PD showed a marginal association (*p* = 0.053). For sdsresp2, fewer ADTs (*p* = 0.003) were associated with response. For functional remission, remitters had lower baseline SDS scores (18.15 ± 5.82 vs. 23.00 ± 3.45, *p* = 0.002), fewer ADTs (3.85 ± 1.66 vs. 5.63 ± 2.36, *p* = 0.001), and more frequent clinically ascertained PD comorbidity (*p* = 0.004). Given the clinical ascertainment of PD and the limited subtype-level sample sizes, PD-related findings were considered exploratory.

### Predictors of symptomatic and functional outcome

For secondary functional response endpoints, univariable logistic models were fitted using the baseline variable retained from the univariate screening step. For sdsresp, clinically ascertained PD comorbidity was associated with response (OR = 1.91, *p* = 0.025). For sdsresp2, fewer ADTs were associated with response (OR = 0.78, *p* = 0.005). For Month 6 functional remission, the parsimonious prognostic logistic regression model included baseline SDS and ADTs. Higher baseline SDS and a higher number of ADTs were both associated with lower odds of functional remission (SDS_M0: OR = 0.73, 95% CI: 0.59–0.89, *p* = 0.002; ADTs: OR = 0.53, 95% CI: 0.35–0.82, *p* = 0.004). Bootstrap internal validation supported the stability of these associations, with bootstrap 95% confidence intervals excluding zero for both predictors (Supplementary Table S4). In the sensitivity model, additionally including clinically ascertained PD comorbidity, baseline SDS, and ADTs remained associated with functional remission, whereas PD was not independently associated with this outcome (Supplementary Table S5). Overall, these findings suggest that clinically meaningful functional improvement and full functional remission are partially distinct outcomes: baseline functional burden was most relevant to remission, whereas prior treatment burden was more informative for functional response, particularly for sdsresp2.

### Treatment-exposure analyses

Median weekly esketamine dose was 84 mg at Month 1, Month 3, and Month 6. The median cumulative dose actually administered across the 6-month observation period, calculated from the baseline-to-M1, M1-to-M3, and M3-to-M6 intervals, was 1008 mg (IQR: 840–1008). When cumulative dosage up to M6 was added to explanatory models, it was not associated with MADRS remission (*p* = 0.724), SDS remission (*p* = 0.642), or sdsresp2 (*p* = 0.130). By contrast, cumulative dosage was significantly associated with sdsresp (*p* = 0.004), indicating that greater overall exposure to esketamine was related to a higher likelihood of clinically meaningful functional improvement. In the same models, PD remained significantly associated with sdsresp (OR = 1.87, *p* = 0.035), whereas baseline SDS remained the dominant predictor of functional remission. These findings suggest that treatment exposure was more closely related to functional improvement than to full remission.

### ROC analysis

**Figure 5.**
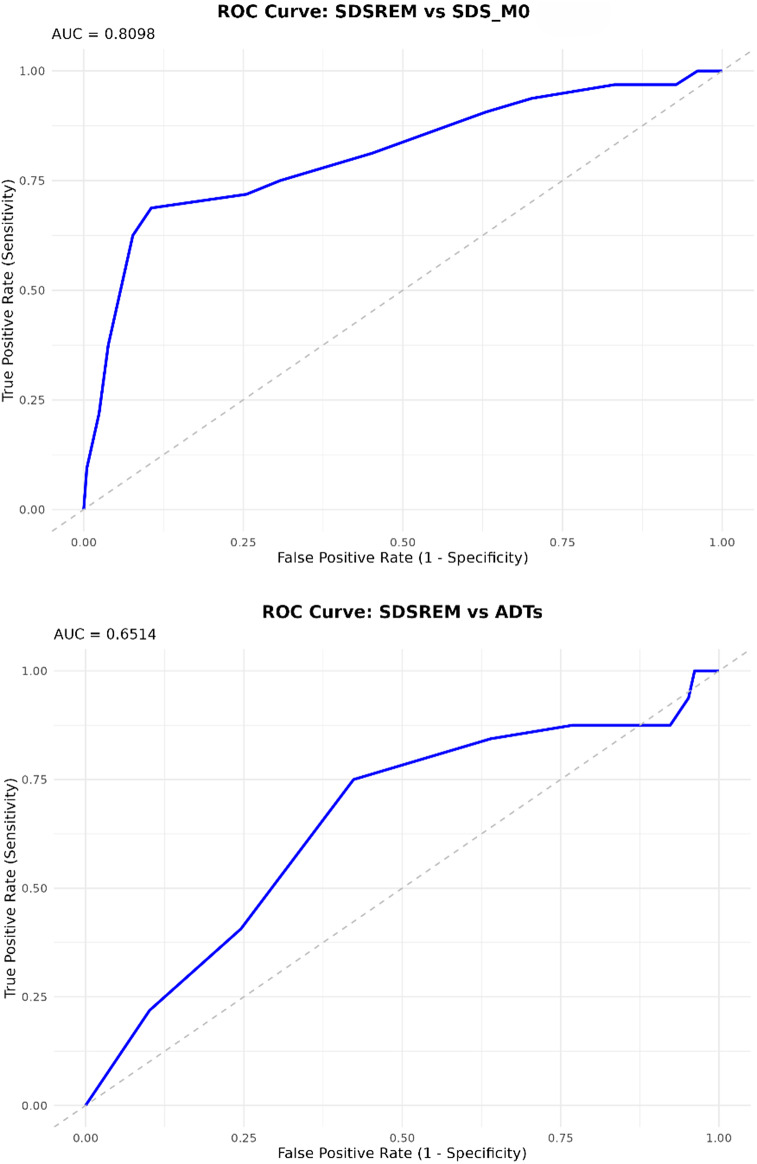
Exploratory ROC curves for baseline variables associated with functional remission at 6 months. Receiver operating characteristic (ROC) curves for baseline SDS and number of previous antidepressant trials (ADTs) in relation to Month 6 functional remission. Functional remission was defined as an SDS total score ≤ 6. (A) The ROC curve for baseline SDS (AUC = 0.8098), and (B) the ROC curve for ADTs (AUC = 0.6514). The diagonal dashed line represents chance discrimination. ROC-derived thresholds were internally evaluated using leave-one-out cross-validation and should be interpreted as sample-dependent estimates rather than clinically validated decision cutoffs. Figure 5. long description.

ROC analyses were restricted to Month 6 functional remission (Figure 5). Baseline SDS and ADTs showed discriminative ability for functional remission, with Youden-derived thresholds of SDS_M0 ≤ 19.5 and ADTs ≤4.5. Because these thresholds were derived from a small sample, leave-one-out cross-validation was performed to evaluate their internal stability. The thresholds were stable across cross-validation iterations; however, cross-validated classification metrics indicated limited performance for individual-level decision-making (Supplementary Table S6). Therefore, these cutoffs should be interpreted as sample-dependent estimates and not as clinically validated decision thresholds.

### Safety and tolerability

Adverse events were recorded during routine clinical monitoring and were generally transient. Adverse events were reported predominantly during the early/acute administration phase rather than during later maintenance administrations. Sedation (10.0%), dissociative symptoms (8.3%), and nausea (6.7%) were the most frequently reported adverse effects. Paresthesias, transient hypertension, and anxiety each occurred in 3.3% of patients, whereas depersonalization, dizziness, urinary incontinence, confusional state, and one brief psychotic episode were each reported in 1.7%. The brief psychotic episode occurred during the early administration phase in a patient with MDD and comorbid Cluster A PD; it resolved within 24 hours under clinical observation and without pharmacological treatment. In total, 43.3% of patients reported at least one adverse effect, while 56.7% reported none.

## Discussion

In this multicenter real-world study, depressive symptoms and functioning improved over 6 months among patients receiving esketamine in routine clinical care. Functional remission accrued gradually across follow-up, suggesting that clinically meaningful functional recovery may continue beyond early treatment assessments. MADRS and SDS changes were moderately correlated, with decreasing correlations over follow-up, supporting partial dissociation between symptomatic and functional recovery.

These findings are consistent with the functional perspective emerging from controlled and real-world esketamine studies, including ESCAPE-TRD, although direct comparisons are limited by differences in design and population [7, 8]. In our cohort, functional remission accrued gradually, increasing from 5.0% at Month 1 to 33.3% at Month 6. This medium-term rate was broadly comparable to the 34.2% functional remission rate reported at Week 32 in the esketamine arm of ESCAPE-TRD, while the early remission rate was lower than the 9.2% reported at Week 4.

Beyond this temporal pattern, our findings also suggest that functional response and functional remission may reflect partly distinct dimensions of recovery. PD comorbidity and cumulative dosage were associated with sdsresp, whereas ADTs were the main correlate of the stricter sdsresp2 endpoint. By contrast, functional remission was most closely related to baseline SDS and ADTs. The association between lower baseline SDS and remission partly reflects the use of an absolute SDS cutoff because patients with lower baseline impairment were closer to the remission threshold. SDS ≤ 6 was retained as the primary remission definition because it is the most supported SDS remission criterion, but its application to a heterogeneous TRD cohort, including BD and PD comorbidity, requires caution. Sensitivity analyses showed that SDS ≤ 5 yielded estimates similar to SDS ≤ 6, whereas SDS ≤ 8 identified a larger subgroup with low residual impairment. ROC-derived thresholds for SDS and ADTs should be viewed as sample-dependent and require external validation before clinical use.

The diagnostic polarity analyses add nuance to the pooled real-world cohort [17–20]. Symptomatic trajectories were broadly comparable between MDD and BD, whereas functional improvement was slower in the BD subgroup. This may indicate greater dissociation between symptom change and functional recovery in bipolar depression. However, the BD subgroup was small, and these findings require replication in larger samples specifically designed to evaluate functional outcomes during esketamine treatment in bipolar depression.

PD-related findings should also be interpreted cautiously. PD was clinically assessed according to DSM-5 criteria by treating psychiatrists, without a structured PD interview, and cluster-level analyses were not feasible. The binary PD variable, therefore, captured heterogeneous personality pathology presentations. Self-reported functional impairment may also be influenced by the ego-syntonic nature of some PD traits [21, 22], despite substantial psychosocial impairment typically observed in clinician-rated assessments [23]. Clinically, the high PD comorbidity rate suggests that integrated psychosocial interventions targeting interpersonal, affective, and functional difficulties may be useful alongside pharmacological treatment.

Treatment-exposure analyses suggested that cumulative dosage was associated with functional response but not remission. This may indicate that greater exposure facilitates clinically meaningful improvement without necessarily leading to full functional recovery. However, in this naturalistic setting, cumulative dose may also reflect clinician-driven treatment continuation among patients showing partial benefit. No unexpected safety signal emerged, and adverse events were generally transient. Overall, these findings support routine functional monitoring alongside symptom scales in TRD. This perspective is also consistent with emerging evidence suggesting that esketamine is not associated with major cognitive worsening in adults with TRD, although the relationship between cognitive change and functional recovery remains to be specifically tested [24].

This study has several limitations. First, the observational design and the absence of a control group preclude causal inference. Improvements cannot be attributed specifically to esketamine and may reflect the natural illness course, regression to the mean, intensified clinical monitoring, treatment expectation, concomitant treatments, or psychotherapy. In addition, changes in concomitant medications during follow-up were not systematically recorded and could not be modeled as time-varying covariates. Second, the sample size was determined by patient availability, and predictor analyses were limited by the number of Month 6 functional remission events. The final prognostic model was, therefore, kept parsimonious and internally assessed using bootstrap resampling, but external validation is required. Third, the inclusion of patients with BD in a current depressive episode reflects real-world practice but introduces diagnostic heterogeneity, and the BD subgroup was too small for robust diagnosis-specific conclusions. Fourth, PD was clinically assessed without structured diagnostic interviews, and subtype-specific analyses were not feasible. Fifth, SDS is a self-report measure and does not capture objective or performance-based functioning. Finally, although interval-censored remission timing was addressed using the Turnbull estimator, temporal precision remains limited by the discrete assessment schedule. Despite these limitations, the study has several strengths. These include the multicenter real-world design, the 6-month follow-up, the explicit focus on functional outcomes, the separation of response and remission analyses, and the use of clinically interpretable models to examine baseline and treatment-related correlates of functional recovery. Together, these features provide a clinically relevant characterization of medium-term functional trajectories during esketamine treatment in a heterogeneous TRD population.

## Conclusion

In this multicenter real-world study, depressive symptoms and functioning improved progressively over 6 months among patients with TRD receiving esketamine in routine clinical care. Functional remission emerged more slowly than symptomatic improvement and continued to accrue throughout follow-up, supporting the clinical value of medium-term functional monitoring. Baseline functional burden and prior treatment burden were the main correlates of functional remission, whereas cumulative esketamine exposure appeared more closely related to functional response. These findings support monitoring functioning alongside symptoms as a distinct clinical outcome in TRD.

## Supporting information

10.1192/j.eurpsy.2026.12233.sm001 Guglielmo et al. supplementary material Guglielmo et al. supplementary material

## Data Availability

Data will be made available upon request.

## Supplementary material

The supplementary material for this article can be found at http://doi.org/10.1192/j.eurpsy.2026.12233.

## Long descriptions

### Figure 1 Long description

The flowchart begins at the top with a central box: Patients screened for study participation across two sites n equals 80. This includes Pavia n equals 46 and Genoa n equals 34.

Two arrows point downward to the next level:

- To the left, a box for Not included n equals 15. This includes Pavia n equals 10 and Genoa n equals 5. Reasons listed are Clinical exclusion criteria n equals 3 (Pavia n equals 2 and Genoa n equals 1) and Declined to participate n equals 12.

- To the right, a box for Included at baseline n equals 65. This includes Pavia n equals 36 and Genoa n equals 29.

From the Included at baseline box, a downward arrow leads to: Included in final analyses n equals 60. This group has Complete Month 6 clinical and functional data, consisting of Pavia n equals 31 and Genoa n equals 29.

A leftward arrow from the final analysis box points to: Treatment not initiated n equals 5. This includes Pavia n equals 5 and Genoa n equals 0.

Finally, a downward arrow from the Treatment not initiated box leads to the bottom-most box: Discontinuation due to adverse events n equals 0.

Navigate back to Figure 1.

### Table 1 Long description

The table contains two columns: Variable and Value.

* Participants, n: 60.

* Age, years, mean S D: 49.8 open parenthesis 15.9 close parenthesis.

* Age range, years: 19 to 80.

* Female sex, n percent: 38 open parenthesis 63.3 close parenthesis.

* University degree, n percent: 16 open parenthesis 26.7 close parenthesis.

* Married, n percent: 33 open parenthesis 55.0 close parenthesis.

* Employment status: Employed 33 open parenthesis 55.0 percent close parenthesis, Unemployed 12 open parenthesis 20.0 percent close parenthesis, Retired 15 open parenthesis 25.0 percent close parenthesis.

* Diagnosis: Major depressive disorder 45 open parenthesis 75.0 percent close parenthesis, Bipolar disorder current depressive episode 15 open parenthesis 25.0 percent close parenthesis.

* Functional impairment at baseline, mean S D: 21.4 open parenthesis 4.9 close parenthesis.

* Depressive symptom severity at baseline, mean S D: 34.2 open parenthesis 7.1 close parenthesis.

* Duration of current depressive episode, months, median I Q R: 12 open bracket 6 to 18 close bracket.

* Duration of mood disorder, years, median I Q R: 20 open bracket 9 to 28 close bracket.

* Previous pharmacological trials, median I Q R: 5 open bracket 3 to 6 close bracket.

* Baseline psychiatric medications: Antipsychotics 29 open parenthesis 48.3 percent close parenthesis, Mood stabilizers 22 open parenthesis 36.7 percent close parenthesis, Benzodiazepines 27 open parenthesis 45 percent close parenthesis.

* Median number of psychopharmacological agents, median I Q R: 3 open bracket 2 to 4 close bracket.

* Ongoing psychotherapy, n percent: 19 open parenthesis 31.7 close parenthesis.

* Psychiatric comorbidity: Personality disorder 32 open parenthesis 53.3 percent close parenthesis, Obsessive-compulsive disorder 1 open parenthesis 1.6 percent close parenthesis, Eating disorder 1 open parenthesis 1.6 percent close parenthesis, Anxiety disorder 1 open parenthesis 1.6 percent close parenthesis.

Navigate back to Table 1.

### Table 2 Long description

The table consists of five columns: Time, Mean, S E, 95 percent C I, and p-value versus M 0.

* At Time M 0 (baseline): Mean is 34.200, S E is 0.917, and 95 percent C I is 32.40 to 36.00.

* At Time M 1: Mean is 23.100, S E is 1.330, 95 percent C I is 20.49 to 25.71, and the p-value is less than 0.001.

* At Time M 3: Mean is 14.800, S E is 1.356, 95 percent C I is 12.14 to 17.46, and the p-value is less than 0.001.

* At Time M 6: Mean is 11.300, S E is 1.046, 95 percent C I is 9.25 to 13.35, and the p-value is less than 0.001.

The data shows a continuous downward trend in depression rating scores over the six-month period. M A D R S stands for Montgomery-Åsberg Depression Rating Scale.

Navigate back to Table 2.

### Table 3 Long description

The table consists of five columns: Time, Mean, S E, 95 percent C I, and p-value versus M 0.

* Row 1: Time M 0, Mean 21.400, S E 0.633, 95 percent C I 20.16 to 22.64, p-value is not applicable.

* Row 2: Time M 1, Mean 18.300, S E 0.736, 95 percent C I 16.86 to 19.74, p-value is less than 0.001.

* Row 3: Time M 3, Mean 12.500, S E 0.852, 95 percent C I 10.83 to 14.17, p-value is less than 0.001.

* Row 4: Time M 6, Mean 10.600, S E 0.891, 95 percent C I 8.85 to 12.35, p-value is less than 0.001.

The data shows a continuous decrease in the mean S D S total score over the six-month period. S D S stands for Sheehan Disability Scale.

Navigate back to Table 3.

### Figure 3 Long description

The horizontal X axis is labeled Timepoint with four categorical markers: M 0, M 1, M 3, and M 6. The vertical Y axis is labeled S D S (Mean plus or minus 95 percent C I) with numerical increments of 12, 16, 20, and 24.

Data flow from left to right:

* At M 0, the mean score is approximately 21.5 with error bars extending from 20 to 23.

* At M 1, the mean score drops to approximately 18.5 with error bars from 17 to 20.

* At M 3, there is a sharper decline to a mean of approximately 12.5 with error bars from 11 to 14.

* At M 6, the mean reaches its lowest point at approximately 10.5 with error bars from 9 to 12.

A solid blue line connects these points, showing a continuous downward trend. A light blue shaded area follows the path of the line, representing the confidence interval band across the follow-up period.

Navigate back to Figure 3.

### Figure 4 Long description

The x-axis is labeled Months from treatment initiation with markers at M 0, M 1, M 3, and M 6. The y-axis is labeled Cumulative probability of functional remission with a scale from 0.00 to 0.45.

Data flow:

* From M 0 to M 1, the probability is 0.00.

* At M 1, the line steps up to 0.05 or 5.0 percent, with a light blue shaded confidence interval band extending from approximately 0.02 to 0.14.

* At M 3, the line steps up to 0.15 or 15.0 percent, with the shaded band extending from approximately 0.08 to 0.26.

* At M 6, the line steps up to 0.333 or 33.3 percent.

Below the graph, a table provides the following values:

* No. at risk: 60 at M 0, 57 at M 1, 51 at M 3, and 40 at M 6.

* New remissions: 0 at M 0, 3 at M 1, 6 at M 3, and 11 at M 6.

* Cum. remitters: 0 at M 0, 3 at M 1, 9 at M 3, and 20 at M 6.

Navigate back to Figure 4.

### Figure 5 Long description

A two-panel vertical stack of Receiver Operating Characteristic line graphs. Both panels share identical axes. The Y axis represents True Positive Rate Sensitivity from 0.00 to 1.00. The X axis represents False Positive Rate 1 minus Specificity from 0.00 to 1.00. A diagonal dashed gray line extends from the origin 0,0 to the top-right 1,1 representing chance discrimination.

Top Panel. Title: R O C Curve S D S R E M versus S D S underscore M 0. An A U C value of 0.8098 is noted at the top left. The solid blue curve rises steeply from the origin, reaching a True Positive Rate of approximately 0.70 at a False Positive Rate of 0.10. It then continues with a shallower positive slope, bowing significantly toward the top-left corner above the chance line.

Bottom Panel. Title: R O C Curve S D S R E M versus A D T s. An A U C value of 0.6514 is noted at the top left. The solid blue curve rises more gradually than the top panel, following a path closer to the diagonal chance line. It reaches a True Positive Rate of 0.75 at a False Positive Rate of 0.45 before plateauing and eventually reaching the top-right corner.

Navigate back to Figure 5.
