# Supplementary material for: Functional outcomes with esketamine in treatment-resistant depression: A 6-month multicenter real-world study
Source: Eur Psychiatry. 2026 Jun 10;69(1):e67. doi: 10.1192/j.eurpsy.2026.12233 (PMC13358991; doi:10.1192/j.eurpsy.2026.12233)
Supplement: Guglielmo et al. supplementary material [file S0924933826122330sup001.docx]

**Supplementary Information**

**Journal: EUROPEAN PSYCHIATRY**

**Functional outcomes with esketamine in treatment-resistant depression: A 6-month multicenter real-world study**

Riccardo Guglielmo^1,2*^, Miriam Olivola^3,4*^, Alberto Inuggi^2^, Elisa Cavanna^1^, Elisa Briasco^1^, Beatriz Pereira da Silva^1,2^, Andrea Escelsior^1,2^, Gabriele Giacomini^2^, Giovanni Martinotti^5,6^, Bernardo Maria Dell’Osso^4,7,8,9^, Mario Amore^1^, Gianluca Serafini^1,2^

^1^Department of Neuroscience, Rehabilitation, Ophthalmology, Genetics, Maternal and Child Health, University of Genoa, Genoa, Italy.

^2^IRCCS Azienda Ospedaliera Metropolitana, Genoa, Italy

^3^Department of Brain and Behavioral Sciences, University of Pavia, Pavia, Italy.

^4^Dipartimento di Salute Mentale e Delle Dipendenze, ASST Fatebenefratelli-Sacco, Milan, Italy.

^5^Department of Neurosciences, Imaging and Clinical Sciences, Università degli Studi G. D’Annunzio, Chieti, Italy

^6^Psychopharmacology, Drug Misuse and Novel Psychoactive Substances Research Unit, School of Life and Medical Sciences, University of Hertfordshire, Hatfield, United Kingdom

^7^Department of Mental Health, Department of Biomedical and Clinical Sciences "Luigi Sacco", University of Milan, Milan, Italy;

^8^"Aldo Ravelli" Center for Nanotechnology and Neurostimulation, University of Milan, Milan, Italy;

^9^Department of Psychiatry and Behavioral Sciences, Stanford University, Stanford, CA, USA

**Supplementary Table S1**. Baseline comparison between treatment initiators and non-initiators

| Variable | Treatment initiators n = 60 | Non-initiators n = 5 | p-value |
| --- | --- | --- | --- |
| Age, years, mean (SD) | 49.8 (15.9) | 55.0 (18.0) | 0.402 |
| Female sex, n (%) | 38 (63.3) | 2 (40.0) | 0.365 |
| Bipolar disorder, n (%) | 15 (25.0) | 0 (0.0) | 0.582 |
| Baseline MADRS, mean (SD) | 34.2 (7.1) | 39.6 (11.1) | 0.330 |
| Baseline SDS, mean (SD) | 21.4 (4.9) | 20.4 (7.8) | 0.961 |
| Previous ADTs, mean (SD) | 5.0 (2.3) | 4.0 (1.2) | 0.331 |
| Disease duration, years, mean (SD) | 20.4 (13.7) | 15.6 (14.4) | 0.475 |
| Current episode duration, months, mean (SD) | 14.6 (12.9) | 18.4 (11.8) | 0.341 |
| Psychiatric comorbidity, n (%) | 39 (65.0) | 3 (60.0) | 1.000 |
| Personality disorder comorbidity, n (%) | 32 (53.3) | 2 (40.0) | 0.663 |
| Ongoing psychotherapy, n (%) | 18 (30.0) | 0 (0.0) | 0.153 |

Continuous variables were compared using Mann–Whitney U tests; categorical variables were compared using Fisher’s exact tests. Comparisons were interpreted descriptively because of the small number of non-initiators. ADTs, antidepressant trials; MADRS, Montgomery–Åsberg Depression Rating Scale; SDS, Sheehan Disability Scale; SD, standard deviation.

**Supplementary Table S2.** Sensitivity analysis of Month 6 functional remission using alternative SDS thresholds

| SDS threshold | N total | N remitters | Remission rate, % | 95% CI, % |
| --- | --- | --- | --- | --- |
| SDS ≤ 5 | 60 | 19 | 31.7 | 21.3–44.2 |
| SDS ≤ 6 | 60 | 20 | 33.3 | 22.7–45.9 |
| SDS ≤ 8 | 60 | 27 | 45.0 | 33.1–57.5 |

Functional remission was evaluated at Month 6 using three SDS thresholds: SDS ≤ 5, SDS ≤ 6, and SDS ≤ 8. SDS ≤ 6 was retained as the primary remission criterion. Confidence intervals were calculated using the Wilson method.

**Supplementary Table S3**. Post-hoc power analysis for longitudinal MADRS and SDS outcomes

| Outcome | Observed standardized effect size | N | Timepoints | Estimated power | Minimum detectable effect size at 80% power |
| --- | --- | --- | --- | --- | --- |
| MADRS | 0.426 | 60 | 4 | 82.7% | 0.469 |
| SDS | 0.452 | 60 | 4 | 86.9% | 0.485 |

Post-hoc power analyses were performed using the achieved sample size and four repeated assessments. Standardized effect sizes are reported in absolute value. Minimum detectable effect sizes refer to 80% power under the assumed repeated-measures correlation structure.

**Supplementary Table S4**. Bootstrap internal validation of the final Month 6 functional remission model

| Predictor | Logistic regression OR | 95% CI | p-value | Bootstrap mean coefficient | Bootstrap SD | Bootstrap 95% CI | Stability |
| --- | --- | --- | --- | --- | --- | --- | --- |
| Baseline SDS | 0.73 | 0.59–0.89 | 0.002 | −0.376 | 0.158 | −0.742 to −0.181 | Stable |
| ADTs | 0.53 | 0.35–0.82 | 0.004 | −0.708 | 0.273 | −1.372 to −0.319 | Stable |

The final prognostic model included baseline SDS and number of previous antidepressant trials (ADTs). Bootstrap internal validation was performed using 1,000 resamples. Coefficients were considered stable when the bootstrap 95% CI excluded zero.

**Supplementary Table S5**. Exploratory sensitivity model for Month 6 functional remission additionally including clinically ascertained PD comorbidity

| Predictor | Coefficient | SE | OR | 95% CI | p-value |
| --- | --- | --- | --- | --- | --- |
| Baseline SDS | −0.290 | 0.102 | 0.75 | 0.61–0.91 | 0.004 |
| ADTs | −0.607 | 0.219 | 0.54 | 0.35–0.84 | 0.006 |
| PD comorbidity | 0.955 | 0.736 | 2.60 | 0.61–11.00 | 0.195 |

This exploratory sensitivity model added clinically ascertained PD comorbidity to the final two-predictor model. Because the inclusion of PD reduced the events-per-predictor ratio below the recommended threshold, results should be interpreted cautiously. PD was not retained in the final prognostic model.

**Supplementary Table S6.** Leave-one-out cross-validation performance of ROC-derived thresholds for Month 6 functional remission

| Metric | Baseline SDS ≤ 19.5 | ADTs ≤ 4.5 |
| --- | --- | --- |
| Accuracy | 0.183 | 0.300 |
| Sensitivity | 0.500 | 0.250 |
| Specificity | 0.025 | 0.325 |
| Positive predictive value | 0.204 | 0.156 |
| Negative predictive value | 0.091 | 0.464 |
| Cross-validated AUC | 0.753 | 0.744 |
| True positives | 10 | 5 |
| False positives | 39 | 27 |
| True negatives | 1 | 13 |
| False negatives | 10 | 15 |

Leave-one-out cross-validation was performed for univariate ROC-derived thresholds. Although the thresholds were stable across iterations, cross-validated classification metrics showed limited individual-level predictive performance. These thresholds should therefore be interpreted as internally evaluated, sample-dependent estimates rather than clinically validated decision cut-offs.
